# RGS2-deficient mice exhibit decreased intraocular pressure and increased retinal ganglion cell survival

**Published:** 2009-03-06

**Authors:** Miyuki Inoue-Mochita, Toshihiro Inoue, David L. Epstein, Kendall J. Blumer, Ponugoti V. Rao

**Affiliations:** 1 Department of Ophthalmology, Duke University School of Medicine, Durham, NC; 2 Pharmacology and Cancer Biology, Duke University School of Medicine, Durham, NC; 3 Department of Cell Biology and Physiology, Washington University School of Medicine, St. Louis, MO

## Abstract

**Purpose:**

Contractile activity of the trabecular meshwork (TM) and ciliary muscle (CM) influences aqueous humor drainage; however, the mechanisms linking tissue contractility and regulation of aqueous humor drainage are not well understood. Regulator of G Protein Signaling 2 (RGS2), a GTPase-activating protein of the Gαq family of proteins, plays a critical role in regulation of contractile activity of vascular smooth muscle and in blood pressure homeostasis. To explore a potential role for RGS2 in intraocular pressure (IOP) homeostasis, we evaluated RGS2 knockout (RGS2^−/−^) mice for changes in IOP.

**Methods:**

IOP was measured using a rebound tonometer in awake male RGS2^−/−^ and littermate wild-type mice. Histological and immunofluorescence analyses were performed to evaluate changes in the iridocorneal structure, actomyosin organization in CM and TM, and retinal ganglion cell survival in both central and peripheral retina.

**Results:**

In repeated measurements, IOP was found to be consistently lower in the RGS2^−/−^ mice compared to littermate wild-type mice. This change in IOP appears to be associated with increased actin filament assembly in the CM, and widening of the Schlemm’s canal in the aqueous humor drainage pathway. Furthermore, ganglion cell number in the central retina was found to be significantly higher in the RGS2^−/−^ mice relative to wild-type mice.

**Conclusions:**

The data suggest that the deficiency of RGS2 decreased IOP, presumably due to increased aqueous humor drainage in association with increased CM contraction. These data indicate a potentially critical role for RGS2 in homeostasis of IOP and for retinal ganglion cell survival.

## Introduction

Glaucoma is a major cause of irreversible blindness worldwide. Elevated intraocular pressure (IOP) due to impaired aqueous humor drainage can cause optic nerve damage and death of retinal ganglion cells, leading to blindness [1,2]. Therefore, there is a great need for understanding the regulation of aqueous humor drainage and finding the molecular targets for novel treatment of increased IOP in glaucoma patients. IOP depends on a balance between aqueous humor secretion from the ciliary body (CB) epithelium and aqueous humor outflow in the trabecular meshwork (TM) [1,3]. In open angle glaucoma patients, it is generally believed that the production of aqueous humor is not increased, but that drainage is impaired due to increased resistance at a level in the outflow pathway close to the inner wall of Schlemm’s canal (SC) [4]. Aqueous humor is drained predominantly through conventional route, consisting of the TM, juxtacanalicular area, and SC [4,5].

Cellular contraction and relaxation properties of both TM and ciliary muscle (CM) have been reported to influence aqueous humor outflow, and thus IOP [3,4,6]. Both TM and CM exhibit smooth muscle-like properties and contain functional muscarinic, α-adrenergic, and β-adrenergic receptors [3,6]. Interestingly, the contractile status of TM and CM has been demonstrated to exert antagonistic effects on aqueous humor outflow, with contraction of the TM leading to decreased aqueous humor outflow, and CM contraction causing increases in aqueous humor drainage [3,4,6]. Moreover, several studies have demonstrated the influence of the Rho GTPase/Rho kinase pathway, myosin II, myosin light chain kinase, and actin polymerization on aqueous humor drainage via the TM outflow pathway [4,7,8]. The Rho/Rho kinase pathway regulates smooth muscle contraction in a calcium-independent manner via myosin II phosphorylation [9]. Agonists of G-protein coupled receptors (GPCR), including angiotensin II, thromboxane A2, lysophosphatidic acid, endothelin-1, and thrombin, induce Rho GTPase activation and myosin light chain (MLC) phosphorylation in TM cells. Perfusion with some of these agonists has been observed to decrease aqueous humor outflow in enucleated eyes [6,10,11], yet inhibition of Rho/Rho kinase and myosin II phosphorylation in the trabecular outflow pathway increases aqueous humor outflow [7]. Taken together, though, these different observations strongly support a potential role for several signaling mechanisms (e.g., GPCR and Rho/Rho kinase-dependent pathways) in the regulation of aqueous humor outflow, the role of these different signaling pathways in mediating the effects of TM and CM contractility on IOP homeostasis is yet to be delineated.

Because TM and CM are smooth muscle-like, we hypothesized that regulator of G-protein signaling 2 (RGS2), a regulatory protein that promotes vascular smooth muscle relaxation and blood pressure homeostasis [12-15], might control IOP. RGS2 is one of more than 20 RGS proteins that regulate signaling via GPCRs in part by accelerating the deactivation rates of Gα subunits via GTP hydrolysis [16,17]. RGS2 preferentially deactivates Gαq [18], which mediates signaling by GPCRs for many important vasoconstrictors including angiotensin II, endothelin-1, and vasopressin signal [19,20]. Activated Gαq in turn triggers smooth muscle contraction via phospholipase C-induced Ca^2+^ release and subsequent increase in MLC phosphorylation by calcium/calmodulin-dependent MLC kinase [21,22]. Gαq can also trigger Rho GTPase signaling by certain Rho exchange factors [21,22]. Homozygous deletion of RGS2 in mice increases blood pressure by impairing the ability of the nitric oxide-cGMP pathway to promote vascular relaxation because inhibition of vasoconstrictor-triggered Gαq signaling is abrogated [14,15].

To explore a potential role of RGS2 and Gαq-coupled GPCR signaling in IOP homeostasis, we examined RGS2 knockout mice (RGS2^−/−^) for changes in IOP in this study. Here we report decreased IOP and increased retinal ganglion cell survival in RGS2^−/−^ mice, possibly a result of increased CM contraction.

## Methods

### Animals

We used the well characterized homozygous RGS2 knockout (RGS2^−/−^) mouse strain along with the littermate wild-type mice obtained from the Blumer laboratory, Washington University School of Medicine, St. Louis, MO [13]. The genetic background of this mouse strain was C57BL/6. Polymerase chain reaction (PCR)-based genotyping was performed using tail DNA to select the RGS2^−/−^ mice. Male RGS2^−/−^ mice 12 to 15 months old and littermate wild-type mice were used in this study. All animal procedures were conducted in accordance with the Association for Research in Vision and Ophthalmology (ARVO) statement for the use of animals in ophthalmic and vision research, under an approved Duke University institutional animal protocol. Animals were maintained under a standard 12 h:12 h light-dark cycle. Food and water were available ad libitum.

### IOP measurement

IOP was measured using a rebound tonometer (Tiolat OY, Helsinki, Finland) according to the manufacturer’s protocol and as described by Wang et al. [23]. Briefly, the wild-type and RGS2*^−/−^* mice were gently restrained in a clear, cone-shaped plastic bag with their heads facing out, and they were tied to the flat plastic stand as described by Wang et al. [23]. Prior to IOP measurements, 0.5% proparacaine anesthetic solution (Bausch & Lomb, Tampa, FL) was applied to the eyes. IOP was measured in both left and right eyes between 11 AM and 4 PM (12 h day light cycle is from 7 AM to 7 PM), and each value was based on an average of five independent tonometer readings. IOP measurements were performed once a week for five weeks on the same animals. Changes in IOP values between the RGS2^−/−^ and littermate wild-type mice were compared at weekly intervals as well as based on the average value of five independent weekly measurements. IOP was expressed per eye, and the sample number was 10 in each group.

### Histology

The mice were euthanized with intraperitoneal injection (150 mg/kg bodyweight) of sodium pentobarbital solution (obtained from Ovation Pharmaceuticals, Inc., Deerfield, IL) and followed by a heart puncture. Eyes were enucleated immediately from both wild-type and RGS2^−/−^ mice and fixed in cold 0.1 M cacodylate buffer, pH 7.3, containing 2.5% glutaraldehyde for at least 2 h. Then the eyes were dissected into anterior and posterior segments and rinsed twice for 15 min with 0.1 M cacodylate buffer. These tissue specimens were postfixed in 0.1 M cacodylate buffer containing 1% osmium tetroxide for 1.5 h at room temperature and rinsed twice for 15 min with cacodylate buffer. The tissue specimens were dehydrated with a series of ethanol (50 to 100%) and propylene oxide, and embedded with Spurr resin. Next 0.8 µm sections, cut with a microtome, were stained with 1% methylene blue, and images were captured using a light microscope (Zeiss Axioplan2, Carl Zeiss Microimaging, Inc., Thornwood, NY). The area and perimeter (total length) of SC in the RGS2^−/−^ and wild-type mice were estimated using Metamorph software (Molecular Devices, Downingtown, PA).

### Immunohistochemistry

Freshly enucleated mouse eyes were initially fixed in 4% paraformaldehyde at 4 °C for 1 h and then dissected into anterior and posterior segments. These tissues were further fixed in 4% paraformaldehyde for 2 h at 4 °C, washed with phosphate buffered saline (PBS, consisting of 1.5 mM KH_2_PO_4_, 2.7 mM Na_2_HPO_4_, and 155 mM NaCl) and cryoprotected in 5% sucrose overnight. The tissue specimens were then shifted to 30% sucrose and incubated overnight before embedding in Optimal Cutting Temperature compound (Tissue-Tek obtained from Sakura Finetek, Torrance, CA) and cryosectioning by cryotome (Microm HM550, Microm, Germany). The tissue cryosections derived from the anterior segment (8 µm) and posterior segment (10 µm) were air dried, washed three times in PBS, and incubated with Image-iT FX Signal Enhancer (Molecular Probes, Eugene, OR) for 30 min. The anterior sections were washed three times in PBS containing 0.3% Triton X-100 for 2 min, and blocked with 3% fetal bovine serum for 2 h at 4 °C. The blocked sections were incubated with phalloidin conjugated with tetra rhodamine isothiocyanate (Sigma-Aldrich, St. Louis, MO) for 20 min at room temperature. Retinal sections (posterior segments) for ganglion cell staining were washed three times in PBS for 2 min each and incubated in 0.5% Triton X-100 for 5 min. After a wash with PBS, the sections were blocked with 10% donkey serum for 2 h at 4 °C and incubated overnight at 4 °C with 1:25 dilution polyclonal Brn-3 primary antibody, which is known to react with Brn-3a, Brn-3b, and Brn-3c subtypes (Cat. No. Sc-6026; Santa Cruz Biotechnology, Santa Cruz, CA). After three 5 min washes with PBS, the specimens were incubated with a 1:100 dilution anti-goat IgG coupled to Alexa-594 (Molecular Probes) for 2 h at room temperature. The tissue sections were washed with PBS for 5 min and co-stained with Hoechst 33258 (Invitrogen, Carlsbad, CA) for 15 min. Slides were mounted with Fluoromount-G (Southern Biotech, Birmingham, AL). Fluorescence images were captured using a Nikon EclipseC90i microscope (Nikon, Tokyo, Japan) and analyzed with Metamorph software.

### Retinal ganglion cell counting

Three retinal cryosections per eye were fixed on each slide, and four slides per eye, from both wild-type and RGS2*^−/−^* mice, were immunostained for Brn-3, a marker of ganglion cells, as described in the previous section. Four individual tissue sections were counted for Brn-3 positive cells. Brn-3 positive cells and the total number of nuclei in the ganglion cell layer of the retina based on Hoechst staining were counted manually per 750 μm of retina, and the values of four independent sections of the same eye were averaged. These cell counts were performed for the central and peripheral retina, and differences in the cell count between the wild-type and RGS2^−/−^ mice were determined.

### Cell culture

To confirm the expression of RGS2 and Gαq in TM and CB cells, we isolated human TM and human CB cells from human donor eyes by digesting the TM tissue with Type IV collagenase as described previously [24]. The cells were cultured in Dulbecco’s Modified Eagle Medium with 10% fetal bovine serum, 100 U/ml penicillin, and 100 μg/ml streptomycin at 37 °C under 5% CO_2_.

### Reverse transcriptase-polymerase chain reaction

Reverse transcriptase-polymerase chain reaction (RT–PCR) analysis was performed on RGS2 and Gαq. Both human TM and CB cells were grown to confluence, and total RNA was extracted using the Rneasy Micro kit (Qiagen, Valencia, CA) according to the manufacturer’s instructions. Total RNA was used for reverse transcription, which was performed with the Advantage® RT-for-PCR kit (Clontech, Mountain View, CA). PCR amplification was performed using the Choice-Taq^TM^ DNA polymerase kit (Denville Scientific, Metuchen, NJ) and the following gene-specific oligonucleotide primers: RGS2: (5′-GGC TGC TTT ACA ACT GCC AGA A-3′/5′-GTT TCC TGA ACA CCC AGG CTG AA-3′) and Gαq: (5′-CCC TGC CTA CCT GCC TAC GCA ACA A-3′/5′-TGT GGC GCA CGT GAA GTG GGA GTA G-3′). Controls having no RT were included. Prior to RT reactions, total RNA was treated with DNase-1 to eliminate DNA contamination.

### Statistical analysis

Results were presented as the mean plus or minus standard error (SEM), and statistical significance was evaluated by the Student’s *t* test. A value of p<0.05 was considered significant.

## Results

To determine the effects of RGS2, a negative regulator of Gαq on IOP homeostasis, we monitored IOP changes in mice with homozygous deletion of RGS2, using a rebound tonometer. These measurements were performed in conscious 12- to 15-month-old mice. IOP was recorded five times on the same animals (both wild-type and RGS2^−/−^) at a weekly interval. The order of IOP measurements between the RGS2^−/−^ and wild-type mice was random.

**Figure 1:**
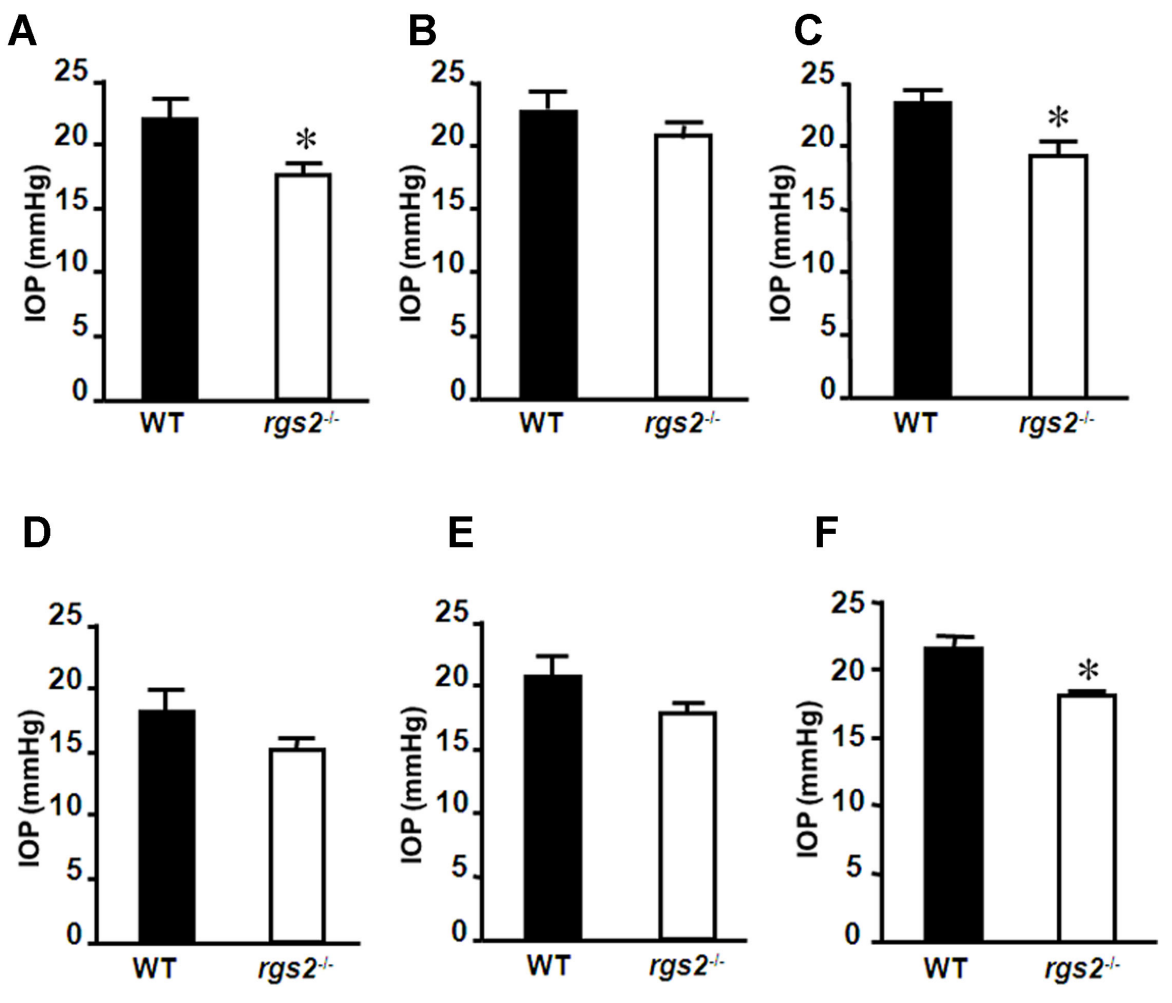
Intraocular pressure changes in the RGS2^−/−^ mice. Diurnal IOP was measured in both right and left eyes in conscious animals, using a rebound tonometer. Measurements were performed five times on the same animals at weekly intervals **A-E**: IOP changes in the RGS2^−/−^ mice were compared with those of wild-type (WT) mice recorded over five different days. **F:** IOP changes between the wild-type and RGS2^−/−^ mice, based on the average of five independent measurements, are shown in panel **A-E**. Each error bar represents the mean±SEM (n=8–10 eyes). Asterisk represents p<0 0.05.

IOP, based on average values of 10 eyes (both left and right), was found to be consistently lower in the RGS2^−/−^ mice compared to the littermate wild-type mice measured over five independent occasions (Figure 1A-E). While the average IOP in wild-type mice was found to be above 20 mmHg (ranging from 18 to 25 mmHg), it was below 18 mmHg (ranging from 14 to 20 mmHg) in the RGS2^−/−^ mice. Additionally, based on the average values of IOP derived from five independent measurements (Figure 1F), IOP was found to be significantly (p<0.05) lower in the RGS2^−/−^ mice (17.98±1.34 mmHg) compared to the wild-type mice (21.39±2.88 mmHg).

**Figure 2:**
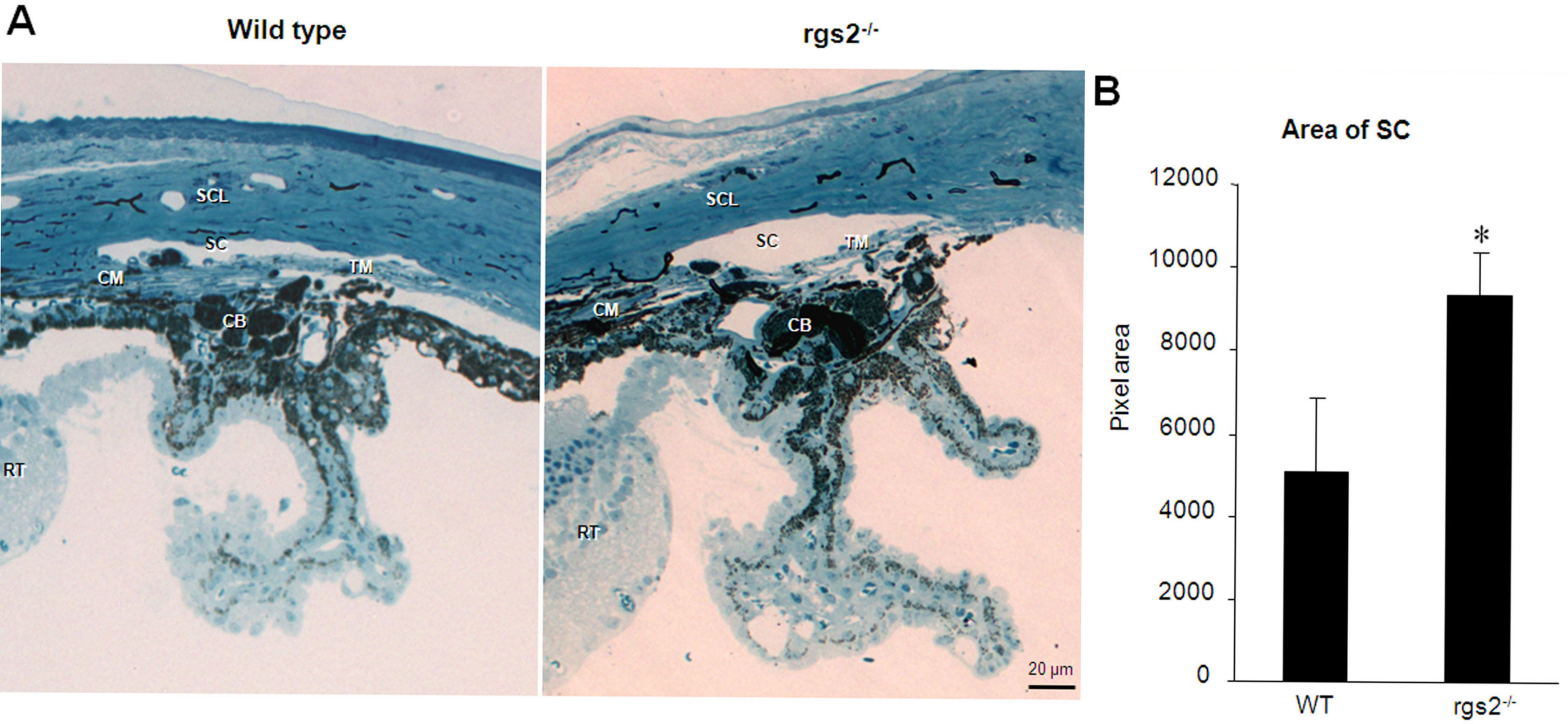
Iridocorneal histological changes in the RGS2^−/−^ mice. **A:** Iridocorneal histological analysis was performed to examine structural differences in the aqueous humor outflow pathway and ciliary body between the RGS2^−/−^ and wild-type mice. The structural integrity of the trabecular meshwork (TM), ciliary body (CB), and ciliary muscle (CM) were found to be comparable between the two groups of mice. However, the Schlemm’s canal (SC) area was found to be significantly larger in the RGS2^−/−^ mice compared to the littermate wild-type mice. **B**: Quantitative changes in the SC area between the RGS2^−/−^ and wild-type mice based on Metamorph pixel analysis. Values are shown as mean±SEM (n=6). Asterisk represents p<0.05. Abbreviations: sclera (SCL), retina (RT).

To gain insight into the IOP changes in the RGS2^−/−^ mice (Figure 1), we performed histologic studies of the iridocorneal angle structure. Figure 2 illustrates the iridocorneal structure of the RGS2^−/−^ and wild-type mice. While there were no obvious differences between the RGS2^−/−^ and wild-type mice in the iridocorneal structure (including TM and CB; Figure 2A), the SC area was found to be 80% larger in the RGS2^−/−^ mice compared to the wild-type mice (based on Metamorph analysis; n=6; Figure 2B). The difference in perimeter (the total length or circumference) of SC between the RGS2^−/−^ and wild-type mice, however, was not significant. The thickness of sclera (in μm) over the aqueous humor drainage angle in the RGS2^−/−^ mice appears to be marginally thinner (78.0±6.8) than in the wild-type mice (90.7±22.4); however, the difference was not found to be significant (p<0.6) between the two groups of mice. The values are based on six samples and expressed as mean±SEM.

**Figure 3:**
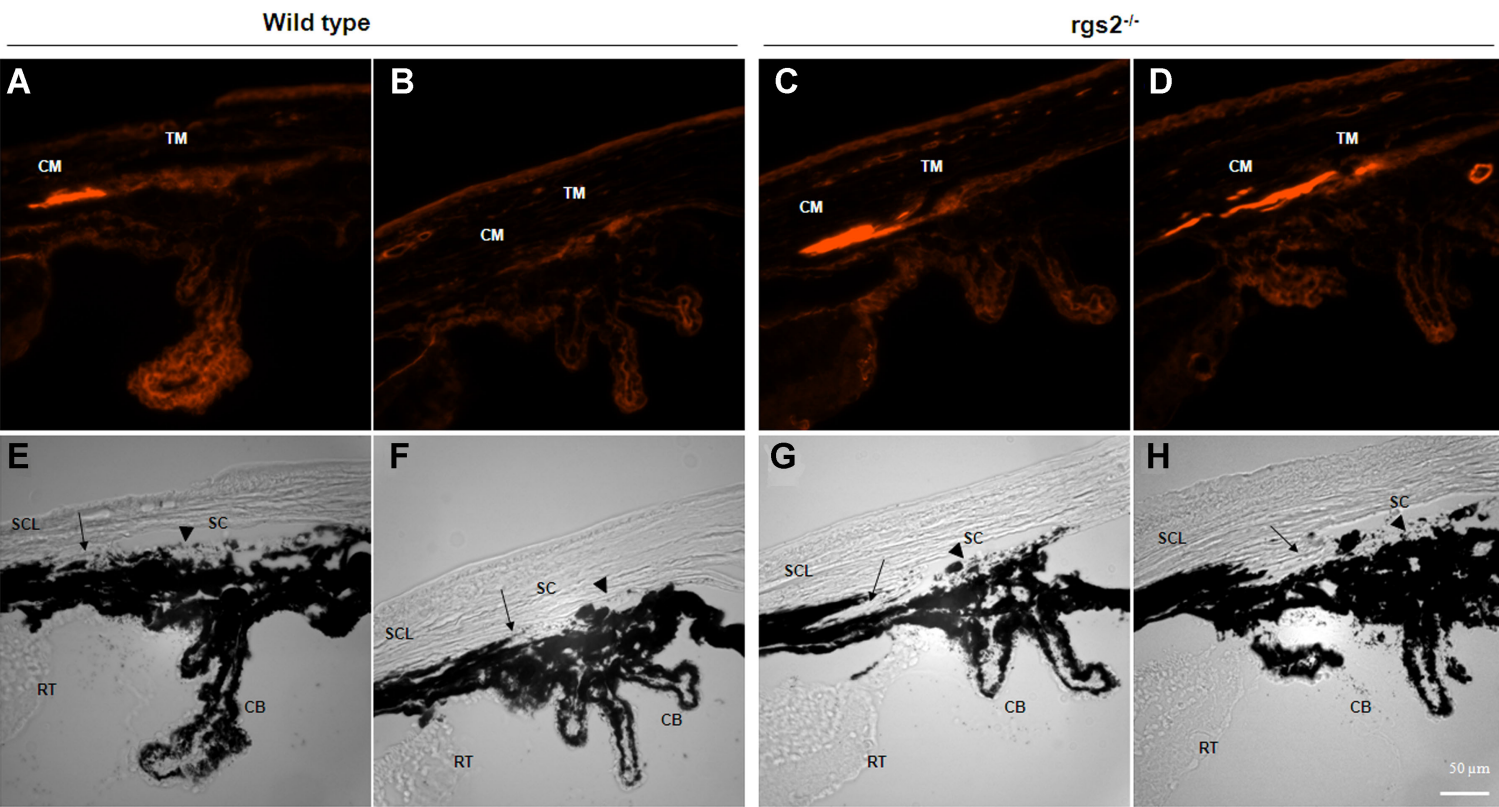
Changes in actin filament staining at the iridocorneal angle of the RGS2^−/−^ mice. Cryosections of the eye anterior chamber including the iridocorneal region derived from the RGS2^−/−^ and wild-type mice were labeled for filamentous actin with rhodamin-phalloidin, and fluorescence images were recorded. Actin filament staining (bright red fluorescence) in the ciliary muscle (CM) and trabecular meshwork (TM) of the RGS2^−/−^ mice (**C** and **D**) was found to be more intense relative to the wild-type mice (**A** and **B**), with much stronger staining in the ciliary body (CB) as opposed to the TM. Panels **E-H** show images of the same specimens stained for the actin filaments (**A-D**), but recorded under bright-field settings. Abbreviations: retina (RT); Schlemm’s canal (SC); sclera (SCL). Scale bar represents 50 µm.

To obtain further insight into the observed increase in SC area in the RGS2^−/−^ mice, we evaluated possible changes in the actin cytoskeletal organization of the iridocorneal angle. The iridocorneal cryosections derived from the RGS2^−/−^ and wild-type mice were stained for actin filaments with rhodamine phalloidin, and the distribution of actin filaments in the iridocorneal angle was recorded using a fluorescence microscope. Interestingly, the specimens of the RGS2^−/−^ mice revealed increased actin filament staining in the CM and TM areas with relatively stronger staining in the CM as compared to the wild-type specimens. Representative images derived from two independent RGS2^−/−^ (Figure 3C,D) and wild-type (Figure 3A,B) mice are shown in Figure 3. Figure 3E-H illustrates the images taken under bright-field of the same sections stained for the actin filament (Figure 3A-D). The results represent the observations based on four independent specimens. The increased actin filament staining in the CM and TM indicates induced actomyosin assembly and contraction, perhaps due to augmented Gαq-dependent GPCR signaling in the RGS2^−/−^ mice.

Primary cultures of human CB and TM cells were confirmed to express both RGS2 and Gαq based on RT–PCR analysis (data not shown). Elevated IOP from impaired aqueous humor drainage has been well recognized to influence the survival status of retinal ganglion cells [25,26]. Therefore, the RGS2^−/−^ mice that showed a significant decrease in IOP compared with wild-type mice (Figure 1F) were evaluated for changes in retinal ganglion cell count. Since glaucoma is an age-related disease, we used 12- to 15-month-old RGS2^−/−^ and wild-type mice to evaluate the chronic effects of RGS2 deficiency on retinal ganglion cell count using tissue cryosections derived from the central and peripheral retina. The retinal tissue cryosections were immunolabeled for Brn-3 (red staining), a well characterized Pit-Oct-Unc (POU) domain protein and extensively used ganglion cell marker [27]. The same tissue specimens were also labeled with Hoechst (blue staining) to count the total number of cells. Both the Brn-3-positive cells and total number of nuclei (Hoechst positive) were manually calculated for the same region and expressed per unit area of the retina.

**Figure 4:**
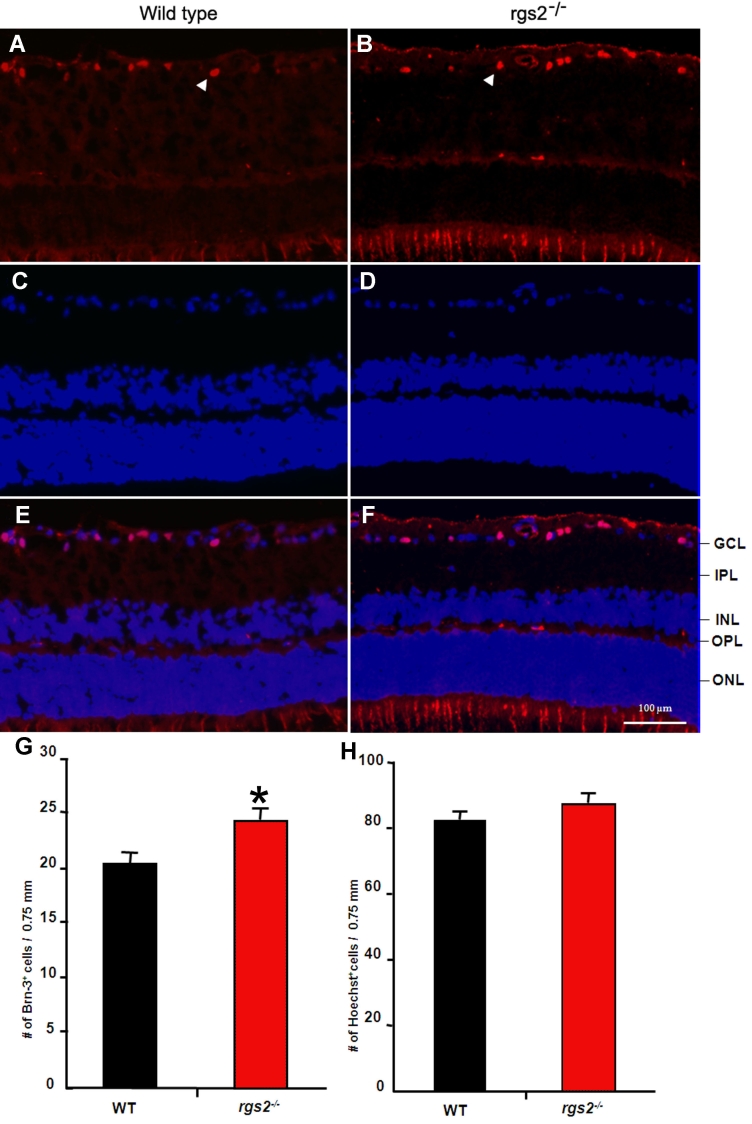
Increased retinal ganglion cell survival in the RGS2−/− mice. To determine the effect of RGS2 on retinal ganglion cell survival in the RGS2−/− mice, we used retinal cryosections derived from 12 to 15-month old male mice and immunolabeled ganglion cells with Brn-3-specific antibody. The same specimens were also stained with Hoechst 33258 dye to count the total number of cells in the ganglion cell region. Panels **A** and **B** show Brn-3-specific immunostaining (red), **C** and **D** show Hoechst staining (blue), and **E** and **F** depict the overlapping of Brn-3 and Hoechst staining. **G:** Quantitative changes in the Brn-3-positive retinal ganglion cells between the RGS2−/− and wild-type mice. **H:** Quantitative changes in the total number of cells in the ganglion cell region of the retina in RGS2−/− and wild-type mice. The RGS2−/− mice show a significant increase in Brn-3-positive cells in the ganglion cell region of the central retina as compared to the wild-type mice. The total number of cells in the ganglion cell region of the central retina based on Hoechst staining did not show significant difference between two groups of the mice. Cell number is expressed per unit area, and values are expressed as mean±SEM (n=4). The asterisk indicates a p<0.05. Abbreviations: ganglion cell layer (GCL); inner plexiform layer (IPL); inner nuclear layer 563 (INL); outer nuclear layer (ONL); outer plexiform layer (OPL). Scale bar represents 100 μm.

Figure 4A-F shows representative fluorescence images of Brn-3-positive cells and Hoechst-positive cells derived from the RGS2^−/−^ and wild-type mice, as well as superimposed images of the Brn-3 and Hoechst labeling. The number of Brn-3-positive cells in the ganglion cell region of the central retina was found to be significantly higher (20% higher than corresponding controls; p<0.05) in the RGS2^−/−^ mice than in the wild-type mice (Figure 4G). In the peripheral retina, however, there was no difference in the Brn-3 positive cells between the two groups of mice. The total number of cell nuclei in the ganglion cell region of the central retina was found to be marginally higher in the RGS2^−/−^ mice but not significantly different from the wild-type mice (Figure 4H). The increased Brn-3-positive cells in the ganglion cell layer of the central retina indicates increased retinal ganglion cell survival in the RGS2^−/−^ mice compared to the littermate wild-type mice.

## Discussion

IOP homeostasis is critically important for both the health of the optic nerve head and retinal ganglion cell survival, and ultimately for normal vision. Increased IOP linked to impaired aqueous humor drainage has been considered a major risk factor for glaucoma [1]. Although various agonists and antagonists of GPCRs have been documented to influence aqueous humor outflow and IOP, very little is known about the identity of predominant GPCR signaling pathways regulating aqueous humor outflow and IOP [3,4,6,10,11,28-30]. To explore the involvement of Gαq-coupled GPCR signaling in IOP homeostasis, we evaluated IOP changes in RGS2 null mice (RGS2^−/−^) in this study. Gαq is a GPCR-coupled G-protein that stimulates the contractile activity of smooth muscle [21,22]. RGS2 serves as a negative regulator of Gαq by accelerating its GTPase activity and thereby terminating the signal response of Gαq [16,17]. RGS2^−/−^ mice showed a decrease in IOP in repeated measurements, and this effect appeared to be associated with an increase in the width of the SC, likely due to increased actomyosin assembly and the resultant increase in tissue contractility in the ciliary muscle.

The mean value of diurnal IOP in the RGS2^−/−^ mice was found to be 17.98±1.34 mmHg compared to 21.39±2.88 mmHg in littermate wild-type mice. Based on several published reports on the rodent glaucoma model, the difference of roughly 3.4 mmHg IOP observed in the RGS2^−/−^ mice is considered to be physiologically significant [31-33]. Moreover, this decreased IOP in the RGS2^−/−^ mice is likely a chronic response stemming from the deletion of RGS2. In this study, the nocturnal IOP was not monitored; however, since nocturnal IOP is higher relative to the diurnal/sleep period in rodents [32,34], it is possible that the lowered IOP (diurnal) observed in RGS2^−/−^ mice is indicative of a much more significant drop in the IOP associated with the nocturnal period.

Interestingly, the lowered IOP in the RGS2^−/−^ mice was found to be associated with an increase in the SC area, owing to a widening of the SC. Although the specific mechanisms responsible for the observed widening of the SC in the RGS2^−/−^ mice are not known (Figure 2), this observation suggests a possible mechanical deformation of SC. Agonists, such as pilocarpine and other miotics, have been shown to cause mechanical deformation of TM and SC due to increased CM contraction, resulting in increased aqueous humor outflow and decreased IOP [5,6,35]. The tendons arising from the anterior tips of the ciliary muscle insert into the TM and well into the subendothelial region adjacent to the inner wall of the SC [36]. Due to this intimate anatomic relationship between the TM and the CM, when CM contraction is induced by pilocarpine and other miotics, it has been shown to cause TM expansion and widening of the SC and ultimately decreasing outflow resistance [35-37]. The muscarinic receptors (M1 and M3) that are coupled to Gαq have been reported to be expressed in the CM and TM [6,28,30,38-40]. Therefore, we speculate that the increased SC area in the RGS2^−/−^ mice might potentially be due to altered geometry of the aqueous outflow pathway in response to increased CM contraction. For SC morphology we used six individual eyes from each group (RGS2^−/−^ and control), and these animals were euthanized in three different batches on different days. We saw distinct and consistent changes in the RGS2 ^−/−^ mice as compared to wild-type mice, thus it is unlikely that these observations represent the result of fixation artifact.

In support of the hypothesis of increased contraction of the CM in RGS2^−/−^ mice, we also noted an increased staining for actin filaments in the CM, indicating induced contractile actomyosin assembly. The TM tissue also exhibited an increase in actin filament assembly based on phalloidin staining; however, F-actin staining in the CM was much higher relative to that noted in the TM (Figure 3). Increased myosin II phosphorylation via sustained activation of Gαq signaling in the RGS2^−/−^ mice was expected to induce actomyosin assembly, leading to enhanced contraction [22,41], and our results of increased actin filament staining in the CM of RGS2^−/−^ mice supported this possibility. Additionally, impaired nitric oxide-cGMP mediated relaxation which is known to occur in RGS2^−/−^ mice might also have contributed significantly to observed increase in CM actomyosin assembly [12,14,15,42-44]. Though additional biochemical studies are necessary to support this contention, the RGS2^−/−^ mice have been reported to develop systemic hypertension due to impaired vascular smooth muscle relaxation [12,13,15,42]. Moreover, it has been reported that the expression and regulation of RGS2 activity plays a critical role in regulation of vascular endothelial contraction and blood pressure homeostasis [45]. Therefore, changes in RGS2 expression in the outflow pathway and ciliary body might also influence IOP.

IOP values obtained in this study were noted to be slightly higher than reported values for the mouse species [23,46], This discrepancy might have stemmed from the lack of use of general anesthesia in this study. The use of anesthetics has been shown to lower IOP significantly [23,47]. However, it also important to note that, unlike the studies reported by Wang et al. [23], the rebound tonometer was not steadied and fixed during the course of acquiring IOP measurements in our studies. Since agitation of mice has been shown to cause elevated IOP [23], any stress or agitation caused by routine physical handling might also be a contributory factor in the higher IOP values obtained in our study.

Both RGS2 and Gαq were confirmed to be expressed in the TM and CM tissues, however, regulation of RGS2 expression in these tissues is not known. Importantly, RGS2 expression has been shown to be influenced by various pharmacological and physiologic agents in different tissues [48-51]. Therefore, in future studies, analysis of RGS2 expression in the CM and TM tissues of glaucomatous and age-matched normal eyes might provide significant insight into the role of RGS2 in the pathophysiology of glaucoma. Since IOP is determined by the balance between aqueous outflow and inflow [3], it is also necessary to exercise caution while interpreting the results of lowered IOP in RGS2 ^−/−^ mice, particularly with regard to whether this response is solely due to increased aqueous outflow through the TM or also partly owing to decreased secretion of aqueous humor by the ciliary epithelium. Episcleral venous pressure is recognized to potentially influence IOP [52]. However, we did not monitor changes in episcleral venous pressure in the RGS2^−/−^ mice. Thus, it remains to be determined whether there was any influence of episcleral venous pressure on the observed changes in IOP in the RGS2^−/−^ mice.

Since elevated IOP has been shown to influence retinal ganglion cell survival [25,26,53,54], we also evaluated changes in the retinal ganglion cell count in the RGS2^−/−^ mice by immunofluorescence analysis using Brn-3 antibody. The ganglion cell count in the central retina was found to be significantly higher in the RGS2^−/−^ mice compared to the littermate wild-type mice. One plausible explanation for the increased ganglion cell survival in the RGS2^−/−^ mice may relate to the decreased IOP in these mice. The RGS2 ^−/−^ mice used in this study were 12 to 15 months of age, and aging alone decreases retinal ganglion cell survival [55]. Moreover, aging is a major risk factor for glaucoma [1]. Aging of C57BL/6 mice has been reported to alter gene expression profiles and to be associated with increased oxidative stress in different ocular tissues [56,57] Therefore, additional studies are required to also resolve the issue of whether the increased RGC count observed in the RGS2^−/−^ mice is specific to aging animals or whether a similar response can be elicited in young animals as well. Decreased IOP in glaucoma patients has been shown to protect ganglion cells, contributing to their survival [58]. However, it is also important to be aware that one cannot rule out the direct influence of Gαq/RGS2 signaling on ganglion cell survival, independent of the effects on IOP. Indeed, the Gαq-coupled M1 muscarinic receptors and their agonists have been shown to increase retinal ganglion cell survival [59]. In contrast to central retina, the retinal ganglion cell count in the peripheral retina was found to be unaltered in RGS2^−/−^ mice and the reasons for this difference between these two regions of the retina is not clear at present. Although we cannot completely rule out the possibility of any developmental changes in the retina of RGS2^−/−^ mouse, based on histological examination of the retina in these mice compared to wild-type mice, we did not notice any distinction between these two groups of mice. Additionally, in the published work, although these mice have been shown to exhibit abnormalities in T-cell activation, anxiety, aggression, and blood pressure, there are no known developmental changes associated with the deficiency of RGS2 [13].

In conclusion, this pilot study reveals the potential importance of RGS2, a negative regulator of Gαq-coupled GPCR signaling, in IOP homeostasis. Decreased IOP, together with the morphological changes noted in the SC and the increased actin filament staining in the CM of RGS2-deficient mice, indicates a critical role for Gαq-mediated signaling in the regulation of contractile activity of the CM and TM, thereby influencing IOP homeostasis.

## References

[1] Weinreb RN, Khaw PT (2004). Primary open-angle glaucoma.. Lancet.

[2] Quigley HA, Broman AT (2006). The number of people with glaucoma worldwide in 2010 and 2020.. Br J Ophthalmol.

[3] Woodward DF, Gil DW (2004). The inflow and outflow of anti-glaucoma drugs.. Trends Pharmacol Sci.

[4] Gabelt BT, Kaufman PL (2005). Changes in aqueous humor dynamics with age and glaucoma.. Prog Retin Eye Res.

[5] Lutjen-Drecoll E (1999). Functional morphology of the trabecular meshwork in primate eyes.. Prog Retin Eye Res.

[6] Wiederholt M, Thieme H, Stumpff F (2000). The regulation of trabecular meshwork and ciliary muscle contractility.. Prog Retin Eye Res.

[7] Rao VP, Epstein DL (2007). Rho GTPase/Rho kinase inhibition as a novel target for the treatment of glaucoma.. BioDrugs.

[8] Honjo M, Tanihara H, Inatani M, Kido N, Sawamura T, Yue BY, Narumiya S, Honda Y (2001). Effects of rho-associated protein kinase inhibitor Y-27632 on intraocular pressure and outflow facility.. Invest Ophthalmol Vis Sci.

[9] Uehata M, Ishizaki T, Satoh H, Ono T, Kawahara T, Morishita T, Tamakawa H, Yamagami K, Inui J, Maekawa M, Narumiya S (1997). Calcium sensitization of smooth muscle mediated by a Rho-associated protein kinase in hypertension.. Nature.

[10] Rao PV, Deng P, Sasaki Y, Epstein DL (2005). Regulation of myosin light chain phosphorylation in the trabecular meshwork: role in aqueous humour outflow facility.. Exp Eye Res.

[11] Mettu PS, Deng PF, Misra UK, Gawdi G, Epstein DL, Rao PV (2004). Role of lysophospholipid growth factors in the modulation of aqueous humor outflow facility.. Invest Ophthalmol Vis Sci.

[12] Tang KM, Wang GR, Lu P, Karas RH, Aronovitz M, Heximer SP, Kaltenbronn KM, Blumer KJ, Siderovski DP, Zhu Y, Mendelsohn ME (2003). Regulator of G-protein signaling-2 mediates vascular smooth muscle relaxation and blood pressure.. Nat Med.

[13] Heximer SP, Knutsen RH, Sun X, Kaltenbronn KM, Rhee MH, Peng N, Oliveira-dos-Santos A, Penninger JM, Muslin AJ, Steinberg TH, Wyss JM, Mecham RP, Blumer KJ (2003). Hypertension and prolonged vasoconstrictor signaling in RGS2-deficient mice.. J Clin Invest.

[14] Osei-Owusu P, Sun X, Drenan RM, Steinberg TH, Blumer KJ (2007). Regulation of RGS2 and second messenger signaling in vascular smooth muscle cells by cGMP-dependent protein kinase.. J Biol Chem.

[15] Sun X, Kaltenbronn KM, Steinberg TH, Blumer KJ (2005). RGS2 is a mediator of nitric oxide action on blood pressure and vasoconstrictor signaling.. Mol Pharmacol.

[16] Heximer SP, Blumer KJ (2007). RGS proteins: Swiss army knives in seven-transmembrane domain receptor signaling networks.. Sci STKE.

[17] Hollinger S, Hepler JR (2002). Cellular regulation of RGS proteins: modulators and integrators of G protein signaling.. Pharmacol Rev.

[18] Heximer SP, Watson N, Linder ME, Blumer KJ, Hepler JR (1997). RGS2/G0S8 is a selective inhibitor of Gqalpha function.. Proc Natl Acad Sci USA.

[19] Wettschureck N, Rütten H, Zywietz A, Gehring D, Wilkie TM, Chen J, Chien KR, Offermanns S (2001). Absence of pressure overload induced myocardial hypertrophy after conditional inactivation of Galphaq/Galpha11 in cardiomyocytes.. Nat Med.

[20] Feuerstein GZ, Rozanski D (2000). G proteins and heart failure: is Galphaq a novel target for heart failure?. Circ Res.

[21] Wettschureck N, Offermanns S (2005). Mammalian G proteins and their cell type specific functions.. Physiol Rev.

[22] Somlyo AP, Somlyo AV (2003). Ca2+ sensitivity of smooth muscle and nonmuscle myosin II: modulated by G proteins, kinases, and myosin phosphatase.. Physiol Rev.

[23] Wang WH, Millar JC, Pang IH, Wax MB, Clark AF (2005). Noninvasive measurement of rodent intraocular pressure with a rebound tonometer.. Invest Ophthalmol Vis Sci.

[24] Song J, Deng PF, Stinnett SS, Epstein DL, Rao PV (2005). Effects of cholesterol-lowering statins on the aqueous humor outflow pathway.. Invest Ophthalmol Vis Sci.

[25] Urcola JH, Hernandez M, Vecino E (2006). Three experimental glaucoma models in rats: comparison of the effects of intraocular pressure elevation on retinal ganglion cell size and death.. Exp Eye Res.

[26] Ji J, Chang P, Pennesi ME, Yang Z, Zhang J, Li D, Wu SM, Gross RL (2005). Effects of elevated intraocular pressure on mouse retinal ganglion cells.. Vision Res.

[27] Gan L, Wang SW, Huang Z, Klein WH (1999). POU domain factor Brn-3b is essential for retinal ganglion cell differentiation and survival but not for initial cell fate specification.. Dev Biol.

[28] Wiederholt M, Schafer R, Wagner U, Lepple-Wienhues A (1996). Contractile response of the isolated trabecular meshwork and ciliary muscle to cholinergic and adrenergic agents.. Ger J Ophthalmol.

[29] Marquis RE, Whitson JT (2005). Management of glaucoma: focus on pharmacological therapy.. Drugs Aging.

[30] Erickson KA, Schroeder A (2000). Direct effects of muscarinic agents on the outflow pathways in human eyes.. Invest Ophthalmol Vis Sci.

[31] Pang IH, Clark AF (2007). Rodent models for glaucoma retinopathy and optic neuropathy.. J Glaucoma.

[32] Savinova OV, Sugiyama F, Martin JE, Tomarev SI, Paigen BJ, Smith RS, John SW (2001). Intraocular pressure in genetically distinct mice: an update and strain survey.. BMC Genet.

[33] Zhou Y, Grinchuk O, Tomarev SI (2008). Transgenic mice expressing the Tyr437His mutant of human myocilin protein develop glaucoma.. Invest Ophthalmol Vis Sci.

[34] Li R, Liu JH (2008). Telemetric monitoring of 24 h intraocular pressure in conscious and freely moving C57BL/6J and CBA/CaJ mice.. Mol Vis.

[35] Grierson I, Lee WR, Abraham S (1978). Effects of pilocarpine on the morphology of the human outflow apparatus.. Br J Ophthalmol.

[36] Lutjen-Drecoll E, Wiendl H, Kaufman PL (1998). Acute and chronic structural effects of pilocarpine on monkey outflow tissues.. Trans Am Ophthalmol Soc.

[37] Rohen JW, Lutjen E, Barany E (1967). The relation between the ciliary muscle and the trabecular meshwork and its importance for the effect of miotics on aqueous outflow resistance. A study in two contrasting monkey species, Macaca irus and Cercopithecus aethiops.. Albrecht Von Graefes Arch Klin Exp Ophthalmol.

[38] Gupta N, Drance SM, McAllister R, Prasad S, Rootman J, Cynader MS (1994). Localization of M3 muscarinic receptor subtype and mRNA in the human eye.. Ophthalmic Res.

[39] Shade DL, Clark AF, Pang IH (1996). Effects of muscarinic agents on cultured human trabecular meshwork cells.. Exp Eye Res.

[40] Gabelt BT, Kaufman PL, Polansky JR (1990). Ciliary muscle muscarinic binding sites, choline acetyltransferase, and acetylcholinesterase in aging rhesus monkeys.. Invest Ophthalmol Vis Sci.

[41] Wettschureck N, Offermanns S (2002). Rho/Rho-kinase mediated signaling in physiology and pathophysiology.. J Mol Med.

[42] Obst M, Tank J, Plehm R, Blumer KJ, Diedrich A, Jordan J, Luft FC, Gross V (2006). NO-dependent blood pressure regulation in RGS2-deficient mice.. Am J Physiol Regul Integr Comp Physiol.

[43] Kamikawatoko S, Tokoro T, Ishida A, Masuda H, Hamasaki H, Sato J, Azuma H (1998). Nitric oxide relaxes bovine ciliary muscle contracted by carbachol through elevation of cyclic GMP.. Exp Eye Res.

[44] Nathanson JA, McKee M (1995). Identification of an extensive system of nitric oxide-producing cells in the ciliary muscle and outflow pathway of the human eye.. Invest Ophthalmol Vis Sci.

[45] Doggrell SA (2004). Is RGS-2 a new drug development target in cardiovascular disease?. Expert Opin Ther Targets.

[46] Morris CA, Crowston JG, Lindsey JD, Danias J, Weinreb RN (2006). Comparison of invasive and non-invasive tonometry in the mouse.. Exp Eye Res.

[47] Johnson TV, Fan S, Toris CB (2008). Rebound tonometry in conscious, conditioned mice avoids the acute and profound effects of anesthesia on intraocular pressure.. J Ocul Pharmacol Ther.

[48] Fatemi SH, Reutiman TJ, Folsom TD, Bell C, Nos L, Fried P, Pearce DA, Singh S, Siderovski DP, Willard FS, Fukuda M (2006). Chronic olanzapine treatment causes differential expression of genes in frontal cortex of rats as revealed by DNA microarray technique.. Neuropsychopharmacology.

[49] Geurts M, Maloteaux JM, Hermans E (2003). Altered expression of regulators of G-protein signaling (RGS) mRNAs in the striatum of rats undergoing dopamine depletion.. Biochem Pharmacol.

[50] Kobori N, Clifton GL, Dash P (2002). Altered expression of novel genes in the cerebral cortex following experimental brain injury.. Brain Res Mol Brain Res.

[51] Homme M, Schmitt CP, Himmele R, Hoffmann GF, Mehls O, Schaefer F (2003). Vitamin D and dexamethasone inversely regulate parathyroid hormone-induced regulator of G protein signaling-2 expression in osteoblast-like cells.. Endocrinology.

[52] Ruiz-Ederra J, Verkman AS (2006). Mouse model of sustained elevation in intraocular pressure produced by episcleral vein occlusion.. Exp Eye Res.

[53] Levkovitch-Verbin H, Harizman N, Dardik R, Nisgav Y, Vander S, Melamed S (2007). Regulation of cell death and survival pathways in experimental glaucoma.. Exp Eye Res.

[54] Huang W, Fileta JB, Dobberfuhl A, Filippopolous T, Guo Y, Kwon G, Grosskreutz CL (2005). Calcineurin cleavage is triggered by elevated intraocular pressure, and calcineurin inhibition blocks retinal ganglion cell death in experimental glaucoma.. Proc Natl Acad Sci USA.

[55] Neufeld AH, Gachie EN (2003). The inherent, age-dependent loss of retinal ganglion cells is related to the lifespan of the species.. Neurobiol Aging.

[56] Ida H, Boylan SA, Weigel AL, Hjelmeland LM (2003). Age-related changes in the transcriptional profile of mouse RPE/choroid.. Physiol Genomics.

[57] Wolf N, Penn P, Pendergrass W, Van Remmen H, Bartke A, Rabinovitch P, Martin GM (2005). Age-related cataract progression in five mouse models for anti-oxidant protection or hormonal influence.. Exp Eye Res.

[58] The Advanced Glaucoma Intervention Study (AGIS) (2000). 7. The relationship between control of intraocular pressure and visual field deterioration.The AGIS Investigators.. Am J Ophthalmol.

[59] Pereira SP, Medina SV, Araujo EG (2001). Cholinergic activity modulates the survival of retinal ganglion cells in culture: the role of M1 muscarinic receptors.. Int J Dev Neurosci.

